# Socioeconomic Factors and Quality of Life Perceived by Parents and Children with Complex Chronic Conditions in Spain

**DOI:** 10.3390/children8100931

**Published:** 2021-10-17

**Authors:** Bibiana Pérez-Ardanaz, María José Peláez-Cantero, José Miguel Morales-Asencio, Concepción Vellido-González, Alberto Gómez-González, Álvaro León-Campos, Laura Gutiérrez-Rodríguez

**Affiliations:** 1 Faculty of Health Sciences, Universidad de Málaga, 29071 Málaga, Spain; bibianap@uma.es (B.P.-A.); gomezgonzalez88@uma.es (A.G.-G.); alvaroleon@uma.es (Á.L.-C.); laura_gr@uma.es (L.G.-R.); 2 Paediatric Palliative Care Unit, Hospital Regional Universitario de Málaga, 29011 Málaga, Spain; pelaez_mariajose@hotmail.com; 3 Instituto de Investigación Biomédica de Málaga (IBIMA), 29010 Málaga, Spain; 4 Case Manager, Hospital Universitario Virgen de las Nieves, 18014 Granada, Spain; concepcion.vellido.sspa@juntadeandalucia.es

**Keywords:** chronic disease, multiple chronic conditions, children, quality of life, socioeconomic factors

## Abstract

Health-related quality of life of children with complex chronic conditions could be affected by sociodemographic factors. Most studies focus exclusively on the parents’ perceptions of quality of life. This study aimed to determine the health-related quality of life of these children, according to their parents and the children themselves. A cross-sectional study was developed on children aged over five years with complex chronic conditions. Health-related quality of life, educational attainment, and social status were evaluated. A total of 101 children were included with a mean age of 10.48 years, and 35.6% were female. The most frequent disease was oncological (28.7%). Children perceived a better health-related quality of life, compared to their parents’ assessment: median difference −8.4 (95%CI: −9.2 to −3.8). Moreover, differences were observed by socioeconomic factors. Parents and children with complex chronic conditions perceive differently the health-related quality of life. Social determinants associate with an uneven perceived quality of life.

## 1. Introduction

In the last decade, there has been a progressive increase in the prevalence of certain chronic, degenerative, and oncological diseases in child populations that threaten and/or limit their lives [1], resulting in frequent hospitalizations and multiple consultations [2]. These circumstances have a substantial impact on children and their families and pose a major challenge to health services, which have traditionally been oriented towards acute care. Health services must be reoriented towards comprehensive, person-centred care and monitoring of outcomes deemed important by the patients, such as health-related quality of life (HRQL) [3,4].

Many instruments are available to evaluate HRQL, generically, as well as some that are specific to certain situations or diseases. In the case of paediatric care for patients with chronic conditions or palliative care needs, generic instruments are more suitable [5], because the diseases are diverse, and such instruments evaluate perceived health and function in multiple spheres of health, as well as the presence of multimorbidity [6].

To date, very few studies have evaluated HRQL in a paediatric population with complex chronic processes, and, of those, most have focused on groups of specific diseases and have performed evaluations in terms of the parents’ perceptions exclusively [7]. There is a well-established association between socioeconomic status (SES) and health. Previous studies highlighted the importance of the interaction between social factors and complexity of chronicity [8]. The conceptual framework for health inequalities developed by the Spanish Commission to Reduce Social Inequalities in Health, differentiates among structural determinants of health inequalities (including social and political context, social status, gender, education, and ethnicity) and intermediary determinants (including occupational status, living conditions, family care, psychosocial factors, and health care system) [9].

Nonetheless, children’s perceptions about their health, and their clinical and sociodemographic profiles have barely been considered from the standpoint of multimorbidity. The evaluation of HRQL in this population can update the individualization of interventions and provide valuable information to nurses in developing a child- and family-centred care approach [10].

To compare perceptions of HRQL of children with complex chronic conditions with those of their parents, according to groups of complex chronic conditions and the time from the diagnosis of their disease, we evaluated the relationship among sociodemographic factors that impact HRQL, according to the parents, and different complex chronic conditions. Some structural determinants of health (gender, education) and one intermediary determinant (occupational status), as well as geographical dispersion of health services in this population group, were taken in to account.

## 2. Materials and Methods

### 2.1. Study Design and Participants

A cross-sectional study was carried out in children with complex chronic conditions.

This study was conducted in Granada (Spain), at Virgen de las Nieves University Hospital, which belongs to the national public health care system. This is the reference hospital for children aged under 18 years (*n* = 170,808). In 2015, the hospital provided 66,382 paediatric consultations for 14,336 children, of whom 42 died [11].

On average, 10,286 children are treated each year at Virgen de las Nieves University Hospital by paediatric medical and surgical services. To detect a standard deviation in PedsQL of 12 points with an alpha of 0.05 and a precision of 3%, and a design effect of 1.3 it was calculated that 74 children would be needed. This sample was overestimated in a 40% (*n* = 103) to cover dropouts [6]. 

The inclusion criteria were children aged between 5 and 18 years with complex chronic conditions (CCC), defined as “any medical condition that can be reasonably expected to last at least 12 months (unless death intervenes) and to involve either several different organ systems or 1 organ system severely enough to require specialty paediatric care and probably some period of hospitalization in a tertiary care centre” [12], who were treated at the hospital and did not present any condition that would reasonably limit understanding, verbal communication, or the ability to provide informed signed consent, during the period June–December 2016. Children were classified according to the Paediatric CCC classification system version 2, updated for ICD-10 and complex medical technology dependence and transplantation [13].

Exclusion criteria were the presence of any condition that would unacceptably limit understanding (such as language), verbal communication, or the ability to provide consent, and the refusal to participate in the study. After reviewing the patient list to determine their eligibility, the research team approached parents in the waiting rooms of the different clinical consultation areas and in the hospitalization wards. One member of the research team explained the study to them and solicited their consent to participate.

### 2.2. Data Collection

Children were recruited from the lists of patients who attended consultations, were admitted to hospitalisation, or were referred by the case management nurse. 

After identifying the participants, they were scheduled for an interview and to fill in the self-administered questionnaires, both by children and parents together. Previously, they received information about the study and were requested for signed informed consent. Both parents and children completed the questionnaire independently. 

A member of the research team extracted all of the data by means of a structured form that was checked daily.

HRQL was measured on the Spanish version of PedsQL Generic Core Scales 4.0. This is a 23-item questionnaire with four major functional domains: physical, emotional, social, and schooling. PedsQL can be administered to parents of children from 2 to 18 years of age and to children themselves, aged 5 years or older. The score range is a 0–100 scale, where 0 indicates the lowest HRQL and 100 the highest. Cronbach’s alpha coefficient for children is 0.83, and 0.86 for parents on the original version. The coefficient is 0.88 in the Spanish general version [13], and it has shown discriminant and construct validity among multiple paediatric conditions [14]. The instrument has been translated and adapted for use in more than 12 countries, including Spain. In our study, the Cronbach’s alpha coefficient obtained was 0.84. The scoring procedure used in our study was the one recommended by the original authors regarding the use of the global score and its domains. In the case of parents, the PEDSQL questionnaire was completed by both parents together, using a unique questionnaire (they discussed the questions and agreed how to score the response). Consequently, the score represented the view of both parents.

According to the framework used in the study, intermediary health determinants were measured by including age (of parents and children), geographic accessibility to health care, educational level, and occupation according to occupational social class [15], together with the medical devices being used.

Duration of disease was evaluated in two ways: parents were asked about when the disease started to manifest, and this information was corroborated by checking the date of the first consultation recorded in the clinical record.

### 2.3. Statistical Analysis

Descriptive and exploratory measures were carried out. The normality of distributions was tested by the Kolmogorov–Smirnov test. Central tendency and dispersion measures are reported as mean (standard deviation) or median (inter-quartile range), depending on the normality of distributions. Bivariate analyses included the following: chi-square test (with Pearson’s chi-square, maximum likelihood ratio, and Fisher’s exact test); Hodges–Lehman’s median difference test, to compare paediatric CCC groups and geographical dispersion of health services with respect to sociodemographic factors, according to the parents; and t student test (or the non-parametric Mann–Whitney’s U for non-normal distributions) to observe HQRL regarding duration of disease. To analyse paediatric CCC, we created the category “others” for groups with less than *n* ≤ 5 that included respiratory, gastrointestinal, hematologic, or immunologic, and renal.

In the case of ANOVA, robust methods were used to evaluate the relation among HRQL, paediatric CCC groups, and sociodemographic factors. Consequently, the homogeneity of variances was evaluated using the Brown–Forsythe approach, which uses medians. Subsequently, post-hoc analyses were carried out by using the Games–Howell test. Spearman correlations were calculated to determine the relationship between HRQL perceived by the children and that perceived by their parents. The level of statistical significance was set at *p* < 0.05. All the analyses were performed with SPSS 25.

### 2.4. Ethical Approval

The study was approved by the Granada Ethics & Research Committee (0655-N-16- A), on 7 July 2016. Consent was obtained both from parents and the children. Previously, they had been informed about the aim of the study. If the children did not agree to participate, they were not included in the study, although their parents could decide whether they wanted to participate. The principles of the Declaration of Helsinki and the relevant standards of good practice were respected.

## 3. Results

### 3.1. Demographics

**Figure 1:**
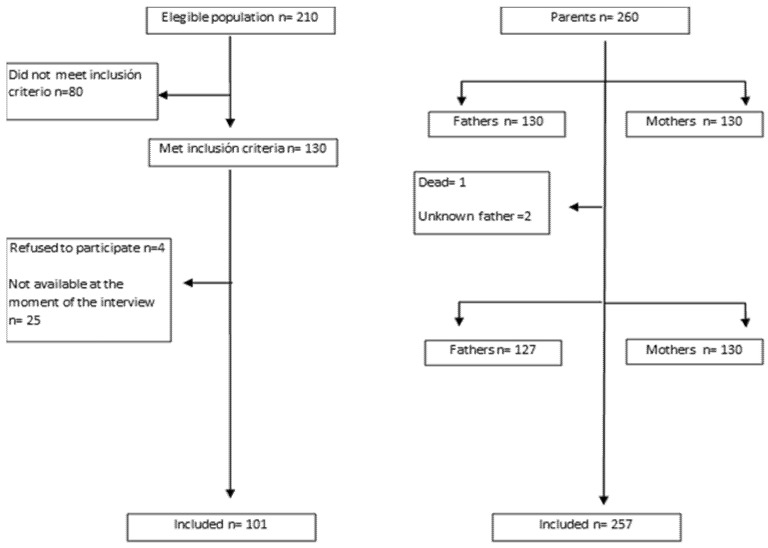
Participants’ flowchart.

A total of 210 family members (including a father, a mother and a child) were invited to participate. The final study sample was composed of 130 family groups (130 children, 130 fathers, and 130 mothers) (Figure 1). From this sample, 29 children did not respond to the PEDSQL questionnaire (either by refusal or they were not available at the time of the interview, because they were at school or in the course of a diagnostic test).

The children’s sample was composed of 65 (64.4%) males and 36 (35.6%) females. Their average age was 10.5 years (SD 3.1), and the majority (90.1%; *n* = 91) were of Spanish nationality. On average, they had the disease for 6.1 (SD 4.6) years.

### 3.2. Characteristics of the Participants

The most frequent types of diseases, according to those with paediatric CCC were malignancy (28.7%; *n* = 29), and other congenital or genetic (17.8%; *n* = 18). 

Some children made chronic use of some form of health technology at home, such as percutaneous gastrostomy (4%; *n* = 4) or vesical catheter (4%; *n* = 4).

A significant proportion of the children (57.4%; *n* = 58) suffered geographical dispersion of health services and were being attended at several centres simultaneously. In addition, 18.8% (*n* = 19) of the children had been referred to Granada from other provinces. Table 1 details the characteristics of the sample.

The majority of fathers had only primary education and were employed in occupations requiring no educational qualification. The educational backgrounds of mothers were similar to those of the fathers, except among those fathers with university degrees (31.5% (*n* = 41) vs. 20.8% (*n* = 27), respectively, and more than half of the mothers were unemployed. No differences were found in the educational level of either the fathers (χ^2^ = 24.8; *p* = 0.052) or the mothers (χ^2^ = 8.3; *p* = 0.910), distributed among the different categories for CCC. Similar findings were obtained for occupational status, (χ^2^ = 13.3; *p* = 0.574 for fathers and χ^2^ = 8.5; *p* = 0.902 for mothers).

We evaluated whether the the geographical dispersion of services could be expressed unevenly among the different occupational or educational levels. Significant variances were found by occupational status of fathers (χ^2^ = 12.7; *p* = 0.005), mothers (χ^2^ = 1.8; *p* = 0.601), and educational level of mothers (χ^2^ = 8.9; *p* = 0.030), although not by fathers’ educational level (χ^2^ = 4.3; *p* = 0.233).

### 3.3. Quality of Life

Overall, the HRQL median scores perceived by the children were higher than those identified by their parents: 65.6 (IQR 22.8) to 57.2 (IQR 17.5), respectively (Hodges–Lehman: −6.5; 95%CI: −9.2, to −3.8). In the physical domain, the scores were 67.2 (IQR 26.6) to 59.4 (IQR 43.7), respectively (Hodges–Lehman: −7.8; 95%CI −10.9, to −3.1). In the psychosocial domain, the scores were 66.7 (IQR 21.2) to 60.0 (IQR 24.2), respectively (Hodges–Lehman: −5.8; 95%CI −9.2, to −3.3).

The correlations between the HRQL scores perceived by the children and by their parents were significantly higher in the physical domain 0.738 95%CI (0.64 to 0.82, respectively) and overall, 0.712 95%CI (0.61 to 0.83, respectively), and somewhat lower in the psychosocial domain 0.682 95% CI (0.53 to 0.75, respectively). 

**Figure 2:**
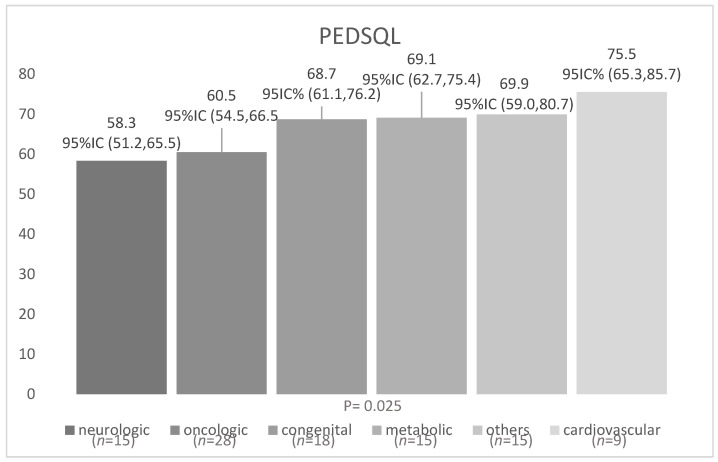
ANOVA of PEDSQL by complex chronic condition groups (perceived by Children).

For HRQL by paediatric CCC group, the ANOVA test showed significant values (F = 2.672; *p* = 0.026) in HRQL perceived by children, although it did not show differences in HRQL perceived by parents (F = 0.520; *p*= 0.761), with those children with neurologic conditions presenting lower HRQL scores relative to the rest of the sample. Nevertheless, the post-hoc comparisons did not show significant differences by paired groups in HRQL perceived by children (Figure 2).

Those parents with higher educational levels and occupational status perceived a better HRQL for their children. Among the fathers, the difference was statistically significant relative to those with only a minimal education level in the summary score: −19.40 95% CI (−36.4, to −2.4); on the psychosocial scale, −18.6 95% CI (−34.8, to −2.5); and −13.27 (−30.8, to 4.2) at primary school. Among the mothers, there were significant differences in the psychosocial domain, −10.1 95%CI (−18.3, to −1.7), in respect to mothers without qualifications. Furthermore, regarding the occupational status, differences were detected in the total score among fathers employed in managerial positions, −17.34 95%CI (−32, to −2.6), and intermediate positions, −11.7 95%CI (−23.3, to −0.1), relative to fathers who were unemployed or retired. In the psychosocial domain, differences were statistically significant for all occupations: managerial, −21 95%CI (−32.9, to −9.1); intermediate, −13.5 95%CI (−24.8, to −2.1); and unqualified, −15.6 95%CI (−26.7, to −4.4); relative to parents who were unemployed or retired (*p* < 0.001). Among the mothers, there were no significant differences by occupational status. No differences were detected in the HRQL perceived by the children, according to the occupational status, or education of their parents (Table 2).

We analysed whether those children who had been diagnosed for a longer time perceived a different HRQL. These analyses were not statistically significant among children: Mann–Whitney’s U: 1436, median difference: −4.35, (95%CI: −10.9, to 2.2); or among parents, Mann–Whitney’s U: 1993, median difference: 0.0001, (95%CI: −6.522, to 6.523).

## 4. Discussion

The aim of this study was to determine HRQL in children with paediatric CCC, according to the perceptions of their parents and of the children themselves, and to explore the relationships bewteen sociodemographic factors influencing HRQL in this population group.

A small number of previous studies have attempted to determine HRQL in paediatric CCC. However, most of these studies focused exclusively on the perceptions of parents or on specific groups of diseases [16]. 

One of the most relevant findings of our study is that HRQL perceived by children is correlated with those evaluated by their parents, although higher scores were obtained in the children’s self-assessments. Some authors recommend that to achieve more reliable measurements, perceptions of HRQL should be obtained from children, as well as their parents, to enable comparison [17], as in our study. Some studies have suggested that parents underestimate the HRQL of their children in certain areas [18], such as emotional or social functioning [17], because their judgements are based on different reasoning processes, styles of response, and interpretations of the items addressed, or because they anticipate a more negative effect of the disability than the child actually experiences [19]. Nevertheless, parents are considered crucial informants for the child and provide vital complementary information, particularly when the child is too small or ill to complete a questionnaire [19]. Notwithstanding, some authors propose a more extreme point of view, and they state that when a child can report their own HRQL, this is preferable to a report from the parents [20,21].

Children with neurological conditions had poorer perceptions of their HRQL. This may arise from an observation of physical changes caused by the progressive deterioration that is a characteristic of this group of diseases [22], or it may be due to the impact of treatment on a physical level. The influence of the instrument used to evaluate HRQL in these findings should be ruled out, since previous research regarding the factor structure of the PedsQL provides confidence that differences in scores obtained between these disease groups are due to actual perceived differences in HRQL rather than differences due to measurement error [23].When comparing chronic conditions, the largest declines of the total PedsQL score can be expected if conditions have severe negative effects on physical function, because the PedsQL assesses this domain with more items than in other domains [24]. Moreover, the largest decline of physical HRQL could be expected among those with abnormal development or impairment in their neurological function such as movement, balance, or posture. Furthermore, school functioning may be particularly negatively affected when many school days are missed, or diseases or side effects of therapy affect cognitive functioning (e.g., epilepsy or traumatic brain injury (TBI)) [25]. Previous studies have shown that young people with neurological disease show the worst HRQL [26,27].

Another important finding of our study is the gradient of higher perceived HQRL among those children whose parents had a higher educational level and occupational status; a large proportion of the mothers in our sample were unemployed, despite having higher education qualifications. Children with chronic conditions impose greater financial burdens [28], greatly heightening the probability of employment difficulties, and this situation usually impacts the mother more severely than the father [29]. Important differences in perceptions of HRQL have been identified among parents and children, according to social class [8,30,31]. This association is well established [32], although correlations among education, income, and occupational class have been reported as moderate and not interchangeable [33], and more research is needed on these measures at the international level [16,34,35]. 

It is noticeable that differences in HRQL by occupational and educational level were obtained in the measures evaluated by the parents, with a clinically important difference between the higher and the lower levels (up to 20 points). By contrast, these differences were diluted when scores were made by children. This finding is significant, as it shows a distorted perception by parents with low occupational and educational level about their children’s health related to quality of life [31]. Thus, these parents may have different expectations and attitudes towards health care providers and services, even towards their children’s daily activities. Nevertheless, this finding would need further research to evaluate the long-term impact of this finding. These results highlight the importance of the interaction between social factors and the complexity of chronicity [8], with the former acting as structural amplifiers of the impact of complex chronic disease [36]. In this sense, the dispersion of services has been reported as a source of stress to family, due to the additional disadvantage of journey, time, and cost, which imposes the additional burden of a charge to the disease [37]. Paediatric nurses, nurse case managers, and community nurses should systematically incorporate the assessment of these dimensions into care planning, due to their influence on the level of complexity [21].

Finally, we observed non-significant perceptions of better HRQL among those children whose disease had been diagnosed for a longer time. This finding has been reported in previous studies [8]. This may be because, in our sample, there was a high proportion of children with chronic complex diseases that were not progressive or long-term, and these patients are a priori stable, or apparently healthy, until they present a critical event or exacerbation of symptoms; or this may reflect adaptation processes (described as the well-being paradox) [38]. On the other hand, some children need hospital care for long periods of time. Their level of care may change, and they may even be discharged from hospital, but this does not mean they are cured, only that a partial improvement or clinical stabilization has been achieved [39].

This study has certain limitations. First, the diagnostic classification system employed may present coding errors by the health professional responsible. Moreover, in our study, to analyse HRQL according to the perceptions of the children and their parents, we did not include children under five years of age, those presenting cognitive impairment, or those who were too ill or unable to complete the questionnaire. Finally, this study is based on cross-sectional data and, therefore, does not consider changes in HRQL associated with the progression of the disease. As, in the present study, there was no control group, we were unable to analyse the differences between such children and the healthy population. Nevertheless, we do not believe that the general level of education in our sample differs from that of parents with healthy children, although their incomes may be lower due to the decreased working time available and the daily healthcare attention that must be provided [40].

Despite these limitations, to our knowledge, this is the first study to provide information on HRQL perceived by parents and their children with CCC.

The findings presented can be extrapolated to the general population of children with CCC, because the underlying characteristics of the parents of children residing in Spain do not differ according to the environment of recruitment, as the Spanish national health system is public, universal, and accessible to all. 

Implications for Practice: patient-reported outcome measures, such as quality of life, must be considered as a key component for the planning and provision of services for children with chronic complexity. Moreover, the incorporation of variables reflecting social determinants is another important element to consider when designing health care services, due to the unequal use currently being made of these services. This may have significant health consequences in the medium to long term.

## 5. Conclusions

Children with CCC present different perceptions of HRQL than those of their parents. Moreover, social determinants, such as educational level, seem to be associated with perceived health-related quality of life. Additionally, the perceived HRQL takes diverse values depending on the CCC group. This finding should be considered when providing care to this population.

On the other hand, the time from the diagnosis seems to have a marginal role in the perceived HRQL. This result invites further exploration in to the coping mechanisms developed by children to adapt to their health status.

A good understanding of the health needs and characteristics of the family environment is essential to facilitate the provision of individualized, comprehensive care, with HRQL as a key measure. To complement these findings, longitudinal studies should be conducted to evaluate the evolution of HRQL over time and to identify the adaptive behaviours of these children and their families.

## Figures and Tables

**Table 1:** Characteristics of the sample (children and parents).

	Children		
	*n* = 101		
	mean (SD) or n (%)		*p*
Age (years) mean (SD)	10.5 (3.1)		
Nationality, n (%)			
Spanish	91 (90.1)		
Other	10 (9.9)		
Duration of the disease (years) mean (SD)	6.1 (4.6)		
**Types of diseases, n (%)**			
Cardiovascular	9 (8.9)		
Respiratory	1 (0.9)		
Renal	5 (4.9)		
Gastrointestinal	4 (3.9)		
Hematologic or immunologic	5 (4.9)		
Metabolic	15 (14.9)		
Congenital or genetic	18 (17.8)		
Neurologic	15 (14.9)		
Oncologic or Malignancy	29 (28.7)		
Dispersion of health care resources, n (%)	106 (59.6)		
Referred from other provinces, n (%)	37 (20.7)		
	Fathers *n* = 127	Mothers *n* = 130	
Age (years) mean (SD)	44.4 (6.7)	41.5 (6.1)	
**Education background, n (%)**			
No qualifications	10 (7.7)	7 (5.4)	(χ^2^ = 117.7) <0.001
Primary education	47 (36.2)	45 (34.6)	
Secondary education	43 (33.1)	37 (28.5)	
University education	27 (20.8)	41 (31.5)	
**Occupation, n (%)**			
Unemployed or retired	23 (17.7)	72 (55.4)	(χ^2^ = 72.2) <0.001
Managerial	17 (13.1)	18 (13.8)	
Supervisory, intermediate level	39 (30)	24 (18.5)	
Unskilled	46 (35.4)	16 (12.3)	

**Table 2:** PEDSQL and sociodemographic characteristics.

	Father (*n* = 130)				Mother (*n* = 130)				Children Comparison with Fathers (*n* = 101) ^†^				Children Comparison with Mothers (*n* = 101) ^††^			
			
			
	Mean (SD)	95%CI		*p*	Mean (SD)	95%CI		*p*	Mean (SD)	95%CI		*p*	Mean (SD)	95%CI		*p*
**Overall PedsQL Score**	Parents: 57.2 (17.5)								Children: 65.4 (15.8)							
**Educational level** §																
No qualifications (*n* = 17)	44.9 (16.9)	32.8	57.0	0.015	49.7 (19.7)	31.5	67.9	0.135	65.7 (12.1)	54.4	76.9	0.466	67.4 (5.4)	58.8	76.0	0.838
Primary school (*n* = 92)	54.7 (16.2)	50.0	59.5		54.7 (17.0)	49.6	59.7		62.0 (17.1)	56.3	67.7		62.3 (18.1)	57.0	69.7	
Secondary school (*n* = 80)	58.2 (19.0)	52.3	64.0		56.2 (18.7)	49.9	62.4		67.7 (14.6)	62.7	72.9		66.4(14.1)	61.2	71.7	
University (*n* = 68)	64.3 (15.5)	58.1	70.4		62.1 (16.0)	57.0	67.1		66.9 (17.6)	58.7	75.2		66.3 (16.0)	60.7	71.9	
**Occupation** §																
Unemployed/retired (*n* = 95)	47.9 (16.4)	40.8	54.9	0.013	54.7 (19.1)	50.2	59.2	0.084	63.4 (20.1)	51.3	75.6	0.695	64.8 (17.2)	60.1	69.4	0.940
Managerial (*n* = 35)	65.2 (13.2)	58.4	72.0		66.4 (12.8)	60.0	72.8		63.1 (16.2)	52.2	74		67.9 (13.7)	59.6	76.1	
Intermediate (*n* = 63)	59.5 (17.1)	54.0	65.0		58.3 (15.8)	51.6	65.0		68.1 (17.2)	62.0	74.2		65.5 (17.2)	58.8	72.2	
Unqualified (*n* = 62)	57.5 (18.7)	52.0	63.0		55.8 (13.4)	48.6	63.0		64.5 (14.0)	60.0	69.0		65.2 (12.6)	56.2	74.2	
**Psychosoc. PedsQL Score**	Parents: 59 (16.7)								Children: 67 (15.2)							
**Educational level** §																
No qualifications (*n* = 17)	47.8 (18.0)	34.9	60.7	0.002	52.4 (18.5)	35.2	69.5	0.002	66.4 (11.1)	56.2	76.6	0.442	69.2 (7.4)	57.4	80.9	0.696
Primary school (*n* = 92)	55.8 (16.0)	51.1	60.5		55.0 (16.2)	50.1	59.9		63.4 (17.6)	57.5	69.3		64.7 (17.5)	58.5	70.8	
Secondary school (*n* = 80)	60.1 (17.3)	54.8	65.5		58.3(18.7)	52.0	64.5		68.9 (12.9)	64.4	73.4		66.2 (14.3)	60.9	71.6	
University (*n* = 68)	66.5 (14.3)	60.8	72.2		65.1 (13.5)	60.9	69.4		68.8 (16.8)	61.0	76.7		69.0 (14.7)	63.8	74.2	
**Occupation** §																
Unemployed/retired (*n* = 95)	46.1 (16.2)	39.1	53.2	<0.001	58.1 (18.2)	53.8	62.4	0.106	61.8 (21.1)	49.0	74.5	0.576	66.7 (16.5)	62.2	71.2	0.756
Managerial (*n* = 35)	67.1 (11.7)	61.1	73.2		66.8 (12.4)	60.6	73.0		65.9 (15.5)	55.5	76.3		69.7 (14.8)	60.8	78.7	
Intermediate (*n* = 63)	59.6 (16.1)	54.4	64.9		59.6 (15.0)	53.2	65.9		69 (15.0)	63.6	74.3		67.0 (12.0)	61.8	72.2	
Unqualified (*n* = 62)	61.7 (16.8)	56.7	66.7		53.2 (14.4)	45.5	60.9		66.4 (14.4)	61.7	71.1		62.7 (17.1)	50.4	74.9	
**Physical PedsQL Score**	Parents: 53.7 (27.6)								Children: 63.41 (23.5)							
**Educational level** §																
No qualifications (*n* = 17)	39.4 (27.8)	19.5	59.2	0.260	44.6 (25.9)	20.6	68.6	0.774	64.3 (26.9)	39.4	89.1	0.621	64.1 (6.5)	53.7	74.4	0.667
Primary school (*n* = 92)	52 (27.6)	44	50		53.1 (28.8)	44.5	61.7		58.2 (26)	49.6	66.7		59.5 (28.3)	49.8	69.3	
Secondary school (*n* = 80)	54.4 (29.3)	45.4	63.4		52.2 (28.1)	42.8	61.5		65.6 (23)	57.6	73.6		66.7 (22.6)	58.3	75.2	
University (*n* = 68)	59.5 (25.5)	49.5	69.4		55.9 (26.9)	47.5	64.3		63.4 (22.7)	52.8	74.1		61.2 (21.6)	53.5	68.9	
**Occupation** §																
Unemployed/retired (*n* = 95)	49.5 (29.6)	36.9	62	0.235	48.4 (28.6)	41.7	55.1	0.093	62.9 (29.5)	45.9	80	0.696	61.2 (24.9)	54.4	67.9	0.948
Managerial (*n* = 35)	60.4 (24.7)	48.1	72.7		65.6 (22.2)	54.6	76.6		57.9 (21.5)	43.5	72.4		64.4 (17.5)	53.8	75	
Intermediate (*n* = 63)	59.4 (26.3)	50.8	67.9		55.4 (27.9)	43.8	66.9		66.6 (25.4)	57.5	75.6		62.7 (27)	51.1	74.4	
Unqualified (*n* = 62)	49.5 (28.4)	41.1	58		57.9 (25.1)	45	70.8		61 (22.1)	53.8	68.1		65.1 (21.8)	50.4	79.7	

^†^ Father educational or occupational level, ^††^ Mother educational or occupational level. § ANOVA.
